# Effects of snack portion size on anticipated and experienced hunger, eating enjoyment, and perceived healthiness among children

**DOI:** 10.1186/s12966-020-00974-z

**Published:** 2020-06-01

**Authors:** Camille Schwartz, Christine Lange, Celia Hachefa, Yann Cornil, Sophie Nicklaus, Pierre Chandon

**Affiliations:** 1 Centre des Sciences du Goût et de l’Alimentation, AgroSup Dijon, CNRS, INRAE, Université Bourgogne Franche-Comté, F-21000 Dijon, France; 2 INSEAD, Sorbonne Université Behavioural Lab., F-77300 Fontainebleau, France; 3 University of British Columbia, Sauder School of Business, Vancouver, Canada

**Keywords:** Portion size, Anticipations, Children, Determinants of portion size choice, Snack food

## Abstract

**Background:**

Large portion sizes encourage overconsumption. Prior studies suggest that this may be due to errors in anticipating the effects of portion size, although the studies were limited to adults and energy-dense foods.

**Objective:**

Our aim was to investigate potential anticipation errors related to the effects of portion size on hunger, eating enjoyment, and healthiness ratings among 8-to-11-year-old children, for snacks differing in energy density and healthiness perception, and as a function of initial hunger.

**Methods:**

In a within-subject design, 83 children aged 8 to 11 years old were first asked to anticipate how much they would enjoy, how hungry they would feel after eating, and how healthy it would be to eat a recommended serving size, a 50% larger portion, and a 125% larger portion of brownie or applesauce. Over six subsequent sessions, the children were asked to eat all of each of these portions and then rate their post-intake enjoyment, residual hunger, and healthiness perceptions. We also measured hunger at the beginning of each session.

**Results:**

For both snacks, larger portions reduced anticipated and experienced residual hunger similarly. In contrast, larger portions increased anticipated but not experienced eating enjoyment for both snacks; although larger portions increased anticipated and experienced enjoyment ratings among extremely hungry children. All children under-anticipated how much they would enjoy the smaller portion sizes. Healthiness ratings were unaffected by portion size for both snacks but differed across foods (applesauce vs. brownie).

**Conclusions:**

Children anticipate the effects of portion size on hunger change accurately, overestimate the effects of portion size on eating enjoyment, and rate food healthiness on food type and not portion size. Helping children better anticipate the enjoyment from smaller (recommended) portion sizes and understand that food quantity, not just quality, matters for healthy eating may be a solution to improve portion control.

## Background

Like adults, children eat more when they are served larger portions, a phenomenon known as the Portion Size Effect (PSE) [1–4]. Portion sizes have increased steadily over the past 30 to 40 years, both in the United States and in Europe, particularly for snacks [5, 6]. In England, for example, the size of bagels increased by 24% between 1993 and 2013 [7]. As a result, public health authorities including the World Health Organization [8] and Public Health England [9] have recommended downsizing the food portions given to children. A meta-analysis found that, of all healthy eating nudges, portion downsizing had by far the greatest impact on consumption decisions [10]. Another recent meta-analysis found that increasing the size of food portions given to children aged 2 to 12 years old by 51–100% led to a 13% increase in daily energy intake (standardized mean difference = 0.47 [95% CI: 0.39–0.55], which means that intake increased by 0.47 standard deviations), for both unit and amorphous foods [3] and also underscored the need for more research to understand the PSE mechanism in children.

Achieving the goal of downsizing portions requires understanding the effect – both anticipated and experienced – of snack portion sizes on the drivers of children’s food choices such as hunger changes from pre- to post-intake, eating enjoyment, and healthiness judgments. Indeed, it may be challenging for parents to follow the recommended portion downsizing if children believe that smaller portions will leave them hungry, will be less enjoyable, and will not be healthier than the large portions they have become accustomed to [11]. Studying the effects of portion size on children’s anticipated and experienced hunger changes, eating enjoyment and healthiness also advances understanding of the mechanisms underlying PSE, which are poorly understood in children [12, 13].

Studies among adults have examined the links between portion size and expectations about residual hunger, healthiness, and eating enjoyment [14, 15]. Unsurprisingly, adults expect to be less hungry after eating larger portions [16]. How much portion sizes influence anticipated, and experienced, residual hunger among children remains an open question, however. Studies among adults have shown that the perceived healthiness of a food portion is only weakly influenced by its size, especially when compared to its content [17, 18]. A few studies in children have shown that they can take into account the perceived healthiness of a food in their food choices [19, 20], but the evidence is sparse and the few available studies were not related to food portion size choice. Finally, there is evidence that adults tend to expect larger portions of palatable foods to increase eating enjoyment [21] – and that this anticipation may be erroneous [22, 23]. In a recent study [24], 343 women were randomly allocated to four groups. One group was asked to rate the *anticipated* eating enjoyment for five increasing portions of brownie, while the other three groups rated their *actual* eating enjoyment after consuming the smallest, middle, or largest portion, respectively. The authors found evidence of misprediction, in the sense that the participants failed to anticipate that smaller portions would be as enjoyable as larger ones, if not more so. This suggests that PSE may be driven, at least in part, by mispredictions, which are common among adults [25, 26]. Supporting the role of mispredictions when judging portion sizes, a recent study among women found that, although they were aware of eating more when served a larger portion, they underestimated by how much they had increased their consumption [27]. Overall, these findings suggest that people are not fully aware of the effects of portion size on their eating behaviors and their judgments.

Two factors, energy density and hunger, are expected to moderate the relationships between portion size and judgements of residual hunger, eating enjoyment, and healthiness. First, Brunstrom and colleagues [28] showed that the expected satiating properties or estimated calorie content increase linearly with energy density only for low energy-dense foods like fruits and vegetables, whereas the relationships for high energy-dense foods reveal an underestimation. This suggests that errors in anticipating the effects of portion size might vary depending on the energy density of the food being evaluated. Second, there is extensive evidence that hunger at the time of the decision influences portion size perception and choice [29–31]. For example, hunger leads people to choose larger portions. It also reduces the extent to which adults choose their portion size based on their expected impact on eating enjoyment and increases their reliance on portion size’s expected impact on their residual hunger after eating the portion [24].

In summary, downsizing portions in children requires first understanding the drivers of children’s portion size perceptions. Yet, we do not know whether children (unlike adults) can accurately anticipate the effects of portion sizes, nor whether the association between anticipations and experiences is similar for foods varying in energy density or whether it varies depending on hunger level. Consequently, we aim to measure the effects of portion size on anticipated (pre-intake) and experienced (post-intake) ratings of residual hunger, eating enjoyment and healthiness among 8-to-11-year-old children for two snacks differing in energy density and perceived healthiness (chocolate brownie and applesauce), taking into account their initial hunger level.

## Methods

The present study set up is a within-subject design with 7 measurements conducted over 7 weeks. Details are presented below.

### Participants

Children aged 8 to 11 from three primary schools in middle-to-upper-class neighborhoods in Dijon (France) were invited to participate in the study. Exclusion criteria were chronic diseases or food allergies. Parents’ permission to let their child participate in the study was requested by the extra-curricular educators of the schools via a consent form. Children’s oral assent to participate was obtained on the first session. The study was approved by the local ethical committee CPP Est I Bourgogne (2016-A00498–43). We aimed to include 80 participants, a sample size based on the observation that in previous studies conducted among children in the same age range, samples of similar size were large enough to detect differences in hedonic evaluations of foods or in food choices for mid-afternoon snacks [19, 32] in the absence of specific data on portion size evaluation. We enrolled 91 children. In each school, participants were split into groups of three to five, each supervised by one adult experimenter. In recognition of their help, the participating schools were given child-care equipment. 10€ were offered to parents for completing the questionnaires.

### Food products

Two foods commonly used for a mid-afternoon snack were chosen [33]. Brossard® chocolate brownie was chosen as the high-calorie, indulgent food (456 kcal/100 g) and Andros® chunky applesauce as the lower-calorie, healthier food (70 kcal/100 g). Their respective nutritional characteristics are detailed in Table 1. These foods were selected based on an existing study, which showed that applesauce is perceived to be healthier than brownie by children from the same age group but is as liked as brownie [34]. A pre-test conducted during an open lab day with 27 children of the same age as those involved in the main study confirmed that both foods were familiar, liked and acceptable afternoon snacks.

**Table 1:** Study foods: energy (kcal) and quantity (g) for each food and each portion

	Portion 1 (recommended serving size)		Portion 2 (× 1.5^a^)		Portion 3 (× 2.25^a^)	
	Quantity (g)	Calories (kcal)	Quantity (g)	Calories (kcal)	Quantity (g)	Calories (kcal)
**Brownie**	32	146	47	219	71	328
**Applesauce**	100	70	150	105	225	157

^a^As a multiple of the recommended serving size. Each portion was 50% bigger than the preceding one

For each food, three portions were selected. Portion 1 corresponded to the serving size recommended by the manufacturer. Portion 2 was 50% bigger than portion 1. Portion 3 was 50% bigger than portion 2 (e.g., for applesauce, the portions were 100 g, 150 g, and 225 g; for details see Table 1). Hence, the gap between portions 2 and 3 (75 g) was 50% bigger than the gap between portions 1 and 2 (50 g). The geometric increase was chosen to span a large size difference with only three possible sizes and to mimic the typical distribution of portion sizes (e.g., sodas are commonly sold in individual containers of 25 cl, 33 cl, and 50 cl).

Prior research [35] has shown that people are more accurate at estimating increases in portion sizes when this increase occurs in only one dimension (either the height, width, or length of the portion) rather than when all three dimensions increase proportionally. Thus, to ensure that the size increase would be accurately perceived by the children, only one dimension of the portions was changed at a time, the length of the brownie and the height of the applesauce in the transparent container. The pre-tests conducted during an open lab day with children also validated these portion sizes as acceptable and ensured that the children had no difficulty distinguishing the portions.

### Study timeline and measurements

Seven mid-afternoon sessions (within-subject design) were conducted in the school canteen during the after-school program, at the time children usually have an afternoon snack (between 4.00 pm and 6.00 pm) over 7 weeks (1 mid-afternoon session each week, see Supplemental Figure 1). At the time of the session, children had not eaten since lunchtime (two lunch services are offered between 11:50 and 13:20, but children eat at the same lunch service each day). The first mid-afternoon session was dedicated to the measure of pre-intake anticipations, and the next six mid-afternoon sessions were dedicated to the post-intake measures. During the first session, the children provided six pre-intake measures (2 foods × 3 portion sizes) for each variable of interest. Half of the children started with the brownie, the other half started with the applesauce. For each food, the portions were presented according to a Latin Square design to balance carryover and order effects. The three portions of each food were presented in a metal box, one at a time, with lids hiding the other two portions (see Supplemental Figure 1). This way, the portions were only visible by the child evaluating them. In order to involve the children in the evaluation tasks, after rating all variables for one food, the children had to indicate which portion among the three they would choose, knowing that they would receive their chosen portion at the end of the session for one of the two foods. At the end of the session, the children were given one of the two foods (determined by a random draw) in their chosen size (half of the children received the brownie, the other half the applesauce).

During the following six sessions (sessions 2 to 7), the children were asked to eat one of the six combinations of the two foods and the three portion sizes entirely before evaluating it. The food (brownie or applesauce) systematically varied from 1 week to the next and the portion size was determined according to a Latin Square design to balance carryover and order effects (portion size was determined at the child level but food type was randomized at the level of groups of 3–5 children who all ate the same food). The portion was placed in the middle of the box used in the first session and the children were instructed to eat it with a spoon without removing it from the box. This way, the portion was only visible to the child eating it. The size of the portion was not explicitly told to the children. The leftovers, if any, were evaluated by weighing the plates/containers before and after consumption using a digital kitchen scale (1 g, Soehnle, Benfeld, Germany) to verify compliance with the instruction to consume the portion entirely.

**Fig. 1:**
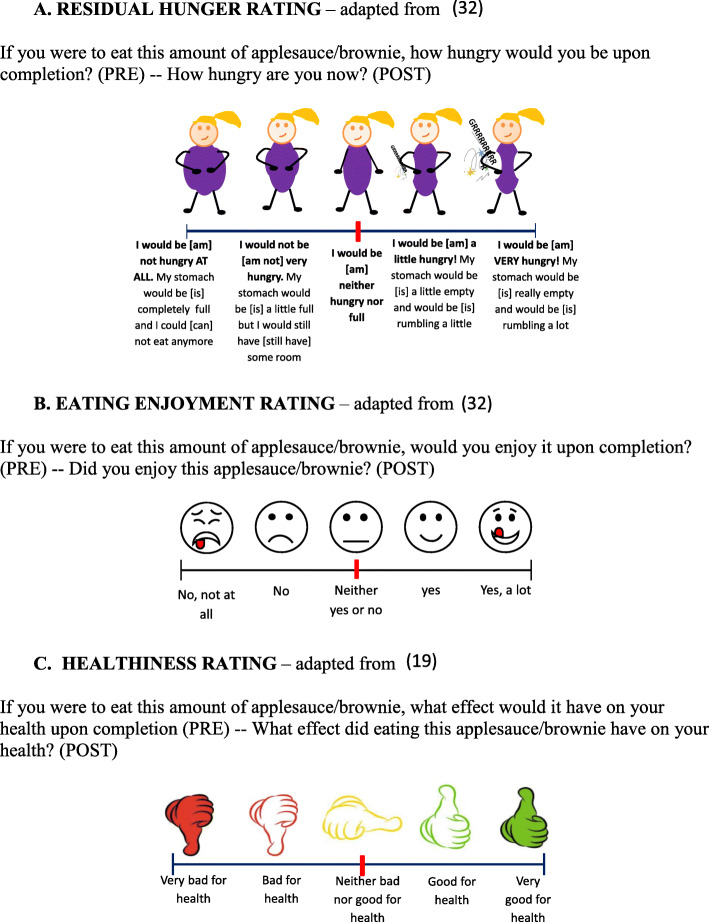
Scales to evaluate residual hunger (**a**), eating enjoyment (**b**), and healthiness (**c**), pre-intake and post-intake. The visual analog scales (0–13.5 cm) were translated from French

Each session started with a self-reported measure of initial hunger. Then, the children were asked to rate their anticipated (session 1) or experienced eating enjoyment (sessions 2 to 7) of the food portion. Next, they rated the anticipated (session 1) or experienced (sessions 2 to 7) healthiness of the food portion. Finally, they reported their anticipated residual hunger after eating the entire portion (session 1) or their experienced residual hunger after eating the assigned food portion (sessions 2 to 7). The same visual analog scales (0–13.5 cm) were used for pre-intake anticipated and post-intake experienced measurements, except that the wording was adapted. Hunger and eating enjoyment were measured with the scales adapted from Lange and colleagues [32], and perceived healthiness was measured with a scale adapted from Marty and colleagues [19]. All these scales have been validated for the studied age range. The scales and their exact wording are presented in Fig. 1.

At the beginning of each of the seven sessions, the same experimenter gave instructions to the children and informed them that, during the session, they could talk to each other about anything except the food they were evaluating. A booklet was provided to each child to write down their answers during each session. The experimenters also explained how to use the three scales, using the instructions provided by the authors of the original scales. After the instructions were given collectively, an experimenter accompanied each group of 3–5 children to explain again the instructions if needed or redirect the discussion if children started to talk about their ratings or about the foods. For all sessions, the experimenter asked the children to remain calm and focused, so that they could win a “silent taster certificate”. This certificate was given to them by the end of the seventh session in exchange for the stars earned by the end of each session, also as a means to thank them for their participation.

At the end of each tasting session (sessions 2–7), children were allowed to draw or engage in other activities to keep them busy until all children had completed their evaluations and/or while the anthropometric measurements were carried out on other children. During sessions 5 and 6, children were weighed to the nearest 0.1 kg using a digital scale (Soehnle, Benfeld, Germany) without shoes. Their height was measured to the nearest 0.1 cm using a stadiometer (Seca, Leicester, Birmingham, UK). All measurements were duplicated and made by an experimenter who had previously been trained by a pediatrician. They were used to calculate body mass index or BMI (weight/height^2^), then converted into age and sex-specific z-scores (z-BMI) based on the French reference data [36].

### Statistical analysis

SPSS version 26.0 was used to conduct statistical analyses. The results are reported as the mean ± SD.

#### Estimation accuracy: anticipated vs. experienced ratings

The goal of the first analyses was to provide baseline results about the level of accuracy of each child’s prediction (for each rating and each portion size) and to examine whether accuracy differed across foods, and for the specific food portion eaten in session 1 (the role of portion size is examined in subsequent analyses). To do so, a two-step procedure was followed. In a first step, each of the 18 pre-intake ratings provided by each child (anticipated eating enjoyment, residual hunger, and perceived healthiness for a small, medium, and large portion of brownie and applesauce) was categorized as 1) an *over-estimation* if it was more than 10% above the corresponding post-intake experienced rating, 2) an *accurate estimation* if it was within 10% of the experienced rating, or 3) as an *underestimation* if it was at least 10% below the experienced rating. In a second step, we predicted with three logistic regressions (one for residual hunger, one for eating enjoyment and one for perceived healthiness) the likelihood of making an accurate prediction based on food type, on a binary variable indicating whether the portion had been eaten by the child in session 1, and the portion size, used here as a covariate. Similar results were obtained when adding sex, age, and z-BMI as covariates and when using a 5% or 15% accuracy threshold (results not shown).

#### Portion size effects on children’s anticipated and experienced ratings

The accuracy analyses previously described show how many children were able to be correct when anticipating their post-intake ratings but do not speak to the role of portion size on children’s anticipated and experienced ratings. In order to analyze portion size effects, we estimated three mixed-level regressions, one per dependent variable (residual hunger, eating enjoyment, and perceived healthiness), combining data from both foods and combining pre-intake (i.e., anticipated) and post-intake (i.e., experienced) ratings.

The key independent variable was portion size, which was coded as − 1 for the smallest portion, 0 for the medium portion, and 1.5 for the large portion. This coding captures the actual size differences between the portions and ensures that the main effect of the variables interacting with portion size is estimated for the medium portion. The other independent variables were a binary variable capturing the difference between anticipated and experienced ratings (Rating: coded as 0.5 for anticipated ratings and − 0.5 for experienced ratings), a binary variable capturing the difference between the two foods (Food: coded as 0.5 for applesauce and − 0.5 for brownie), and all their interactions. Based on prior evidence regarding the effect of hunger [24], the models included initial hunger at the time of the session and its interaction with portion size. Moreover, they all controlled for sex, age, and z-BMI, given that these variables might have an effect on caloric requirements, hence on the ratings. Initial hunger, age and z-BMI were mean-centered. Similar results were obtained without the covariates (not shown here). To account for the fact that each child provided multiple observations, we used mixed-model regressions allowing for correlated errors at the participant level.

When these analyses showed that portion size differently influenced anticipated and experienced ratings (which was the case for eating enjoyment), we used spotlight analyses with two alternate centering of portion size (0, 1, 2.5 and − 2.5, − 1.5, 0 for the small, medium, and large portion, respectively) to estimate the difference between anticipated and experienced ratings for the smallest and largest portion size respectively, rather than for the medium portion size, as done in the main analysis [37]. Another spotlight analysis, in which the “Rating” binary variable was set to 0 for experienced and to 1 for anticipated ratings, was used to estimate the effects of portion size on experienced eating enjoyment, rather than its average effect for both anticipated and experienced eating enjoyment.

Finally, when the interaction initial hunger × portion size was significant, separate models were estimated on two sub-groups of children, based on a median split of their average initial hunger. Given the high level of hunger of all children at the time of the sessions (which were conducted between 4.00 pm and 6.00 pm; *M =* 10.5 ± 1.9 on the 0–13.5 cm visual analog scale for initial hunger), we labeled the group of 42 children with lower hunger level as “hungry” (*M =* 9.0 ± 1.5, t-test of difference from the 6.5 cm scale mid-point = 11.0, *p* < 0.001) and the 41 children with higher hunger level as “very hungry” (*M =* 12.0 ± 0.8, t-test of difference from the 6.5 cm scale mid-point = 46.6, *p* < 0.001).

Supplemental Table 1 provides food and rating-specific coefficients from 12 separate regressions, one for each food and for each of the two ratings (anticipated and experienced) of the three dependent variables (residual hunger, eating enjoyment and perceived healthiness). Additional analyses were conducted to report choice of food portion made by children at the end of the first session, and to evaluate portion size effects at individual level. Their results are available in the Supplemental analyses 1.

## Results

### Participants

Out of the 91 children who agreed to participate, data for both foods were excluded for 8 participants (6 children who had missed the first session, one who had refused to provide answers, and one who was absent twice during sessions 2–7), leaving 83 children. In addition, 6 children refused to consume the applesauce. As a result, data were available for 83 children for the brownie and for 77 children for the applesauce. Sample characteristics are detailed in Table 2. Out of the 83 children, 45 were female (54%). Children were 9.5 ± 0.8 years old and their average weight was 32.0 ± 5.5 kg.

**Table 2:** Characteristics of the participating children (*N* = 83)

	Mean	SD	Min	Max
Age (years)	9.5	0.8	7.9	11.5
Height (cm)	137.8	6.7	124.4	154.4
Weight (kg)	32.0	5.5	22.0	50.1
z-BMI^a^	0.4	1.2	−1.9	4.3

^a^The z-BMI scores were calculated based on age and sex standardized scores for the French population [36]

Children consumed all the portions that they were given (defined as leaving less than 1 g of food) in 77.3% of the cases as they were instructed to do. Out of the 83 children, 27 (32.5%) were categorized as completers, meaning that they never left more than 1 g of food across the six eating occasions, and the rest (56 children, 67.5%) were categorized as non-completers. Comparisons of the two groups are reported in Supplemental Analyses 1.1 and showed that non completers were not statistically different from completers for any of the control variables (initial hunger, z-BMI, age and sex); but they differed for one dependent variable, anticipated eating enjoyment, which was higher for completers (C) than for non-completers (NC) (*M*_*NC*_ = 9.4 ± 2.0 vs *M*_*C*_ = 10.3 ± 1.5, *F* (1,82) = 4.7, *p* = 0.03). Leftovers amounted to 4.5% of the weight of the original portion on average, (2.2% for brownie and 6.9% for applesauce, *F* (1, 479) = 13.3, *p* < 0.01). Analyses of the portion choices made at the end of the first session are reported in Supplemental Analyses 1.2.

### Estimation accuracy: anticipated vs. experienced ratings

**Fig. 2:**
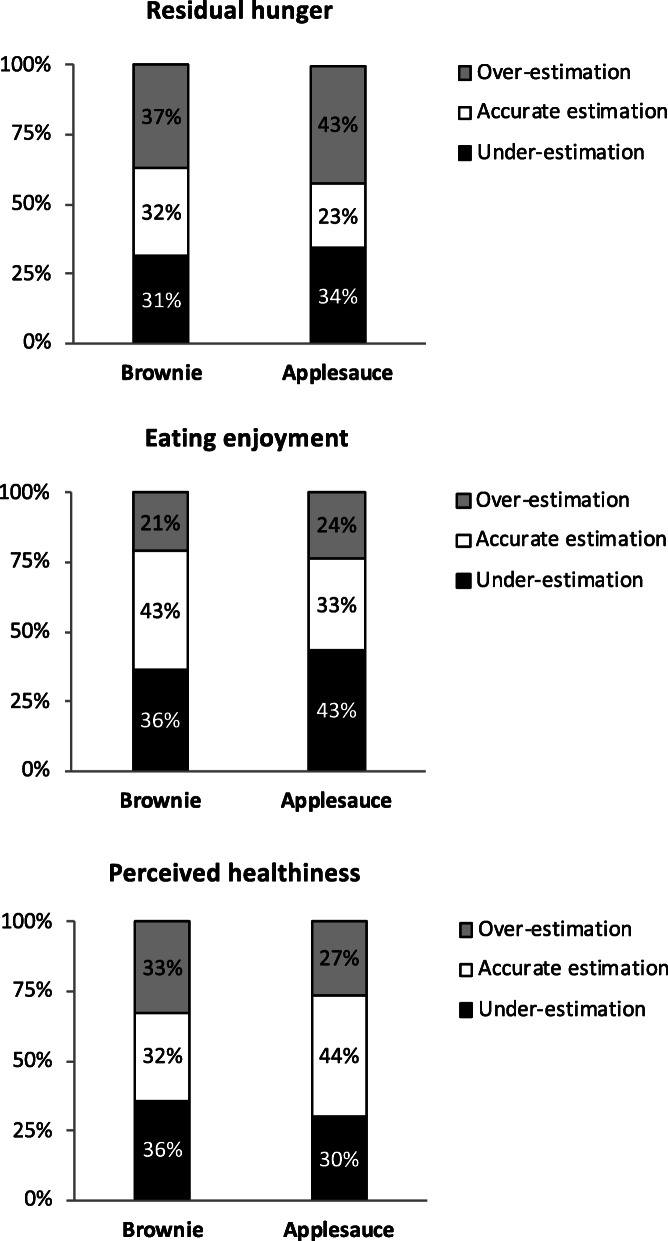
Accuracy of pre-intake anticipations for residual hunger (top), eating enjoyment (middle), and perceived healthiness (bottom). Percentage of over-estimations (more than 10% above the corresponding experienced rating), accurate estimations (within 10% of the corresponding experienced rating), and under-estimations (at least 10% below the corresponding experienced rating) for each food and rating

We found significant differences across foods in the accuracy of pre-intake ratings (*N* = 83 for brownie and *N* = 77 for applesauce, for each variable). Overall, only about one third of the pre-intake ratings were accurate (within 10% of the post-intake ratings), a proportion that varied between 23 and 44% depending on the food and construct (see Fig. 2). Logistic regressions showed statistically significant differences between the two foods in the likelihood of accurate estimations (Wald = 4.24, *p* = 0.040; Wald = 4.01, *p* = 0.045; Wald = 7.34, *p* = 0.007 for residual hunger, eating enjoyment, and perceived healthiness, respectively). Children were more likely to make accurate estimations of residual hunger and eating enjoyment for brownie than for applesauce, but judgements of healthiness were more accurate for applesauce than for brownie.

Furthermore, the estimations made for the portion of food that children had consumed in session 1 were just as accurate as the other estimations (Wald = 0.05, *p* = 0.82; Wald = 2.22, *p* = 0.14; Wald = 0.020, *p* = 0.88 for residual hunger, eating enjoyment, and perceived healthiness, respectively).

### Portion size effects on children’s anticipated and experienced ratings

The accuracy analyses showed that about two-thirds of anticipated ratings were more than 10% off compared than their experienced ratings but are silent about the role of portion size, which is analyzed below.

#### Residual hunger

As shown in Table 3, larger portion sizes decreased residual hunger (B = − 0.76, *t* = − 10.72, *p* < 0.001). Anticipated residual hunger was consistently larger than what was experienced (B = 0.40, *t* = 2.72, *p =* 0.007) but the type of rating did not interact with portion size (B = -0.09, *t* = − 0.65, *p* = 0.52), indicating that portion size similarly reduced anticipated and experienced residual hunger and had the same effect on both foods.

**Table 3:** Separate mixed-level regression coefficients (SE) for each of the three dependent variables

	Residual Hunger		Eating Enjoyment		Perceived Healthiness	
	B	SE	B	SE	B	SE
Intercept	8.28	0.18	9.90	0.18	9.26	0.19
Portion size^a^	-0.76**	0.07	0.34**	0.08	-0.12	0.06
Rating (anticipated vs. experienced)^a^	0.40**	0.15	−0.68**	0.16	−0.09	0.13
Portion size × Rating^a^	−0.09	0.14	0.37*	0.15	−0.02	0.13
Food (applesauce vs. brownie)^a^	−0.24	0.15	−1.29**	0.16	2.50**	0.13
Portion size × Food	0.11	0.14	−0.25	0.15	0.18	0.13
Food × Rating^a^	−0.11	0.29	−0.28	0.32	−0.35	0.26
Portion size × Rating × Food	−0.01	0.28	0.24	0.31	−0.19	0.25
Initial hunger^b^	0.55**	0.04	0.09	0.04	0.06	0.04
Portion size × Initial hunger	0.01	0.03	0.12**	0.03	0.00	0.03
Female	−0.83*	0.36	−0.36	0.35	−0.56	0.38
Age^b^	0.35	0.23	−0.04	0.23	−0.01	0.25
z-BMI^b^	−0.14	0.16	−0.03	0.15	−0.25	0.17

Note: B = unstandardized coefficient of regression. SE = Standard Error. all regressions controlled for child sex, age, and z-BMI. ^a^Portion size was coded as −1 for the smallest portion, 0 for the medium portion, and 1.5 for the big portion; Rating was a binary variable capturing the difference between anticipated and experienced ratings (coded as 0.5 for anticipated ratings and − 0.5 for experienced ratings); Food was a binary variable capturing the difference between the two foods (coded as 0.5 for applesauce and − 0.5 for brownie) ^b^indicates that the variable was mean-centered; ** indicates that the coefficient is statistically different from zero at *p* < 0.01 (* at *p* < 0.05)

**Fig. 3:**
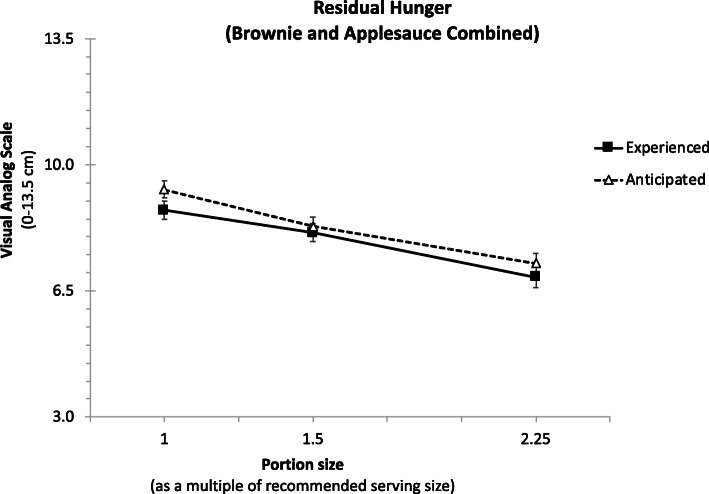
Means anticipated and experienced residual hunger, for both brownie and applesauce. The portion sizes represent the recommended serving size, or 1.5 or 2.25 multiple of this size respectively. Anticipated and experienced residual hunger decreased similarly with portion size, for both foods

Figure 3 plots the mean anticipated and experienced residual hunger for both foods combined. The parallel lines show that the patterns of children’s judgments of how hungry they would feel after imagining eating or actually eating each portion size decreased similarly and linearly with portion size.

There were no statistically significant differences between any of the regression coefficients comparing brownie and applesauce (all *p*’s > 0.11). This showed that portion size influenced anticipated and experienced residual hunger similarly for both foods. Moreover, initial hunger at the time of the session was associated with increased residual hunger (B = 0.54, t = 13.06, *p* < 0.001), independently of portion size (the interaction effect between initial hunger and portion size was insignificant; B = 0.01, *t* = 0.26, *p* = 0.79).

#### Eating enjoyment

**Fig. 4:**
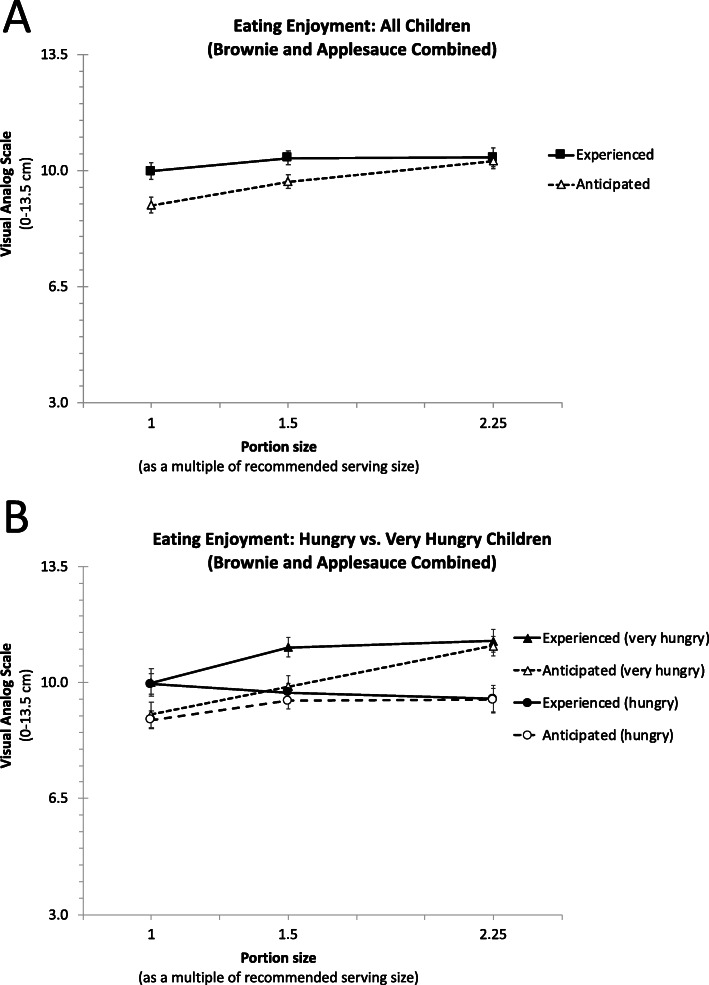
Means anticipated and experienced eating enjoyment for both brownie and applesauce among all children (**a**), very hungry children and hungry children (**b**). The portion sizes represent the recommended serving size, or 1.5 or 2.25 multiple of this size respectively. Overall, children expected eating enjoyment to increase with portion size, whereas experienced eating enjoyment was independent of portion size. For that reason, the eating enjoyment of the smaller two portions (but not of larger ones) were underestimated. Among very hungry children, portion size increased both anticipated and experienced eating enjoyment

Table 3 shows that larger portion sizes increased eating enjoyment overall (B = 0.34, *t* = 4.41, *p* < 0.001), even though anticipated eating enjoyment was consistently below what was experienced (B = -0.68, *t* = − 4.25, *p <* 0.001). Importantly, the interaction effect between portion size and anticipated (vs. experienced) rating was positive and statistically significant (B = 0.37, *t* = 2.38, *p =* 0.018), indicating that larger portion sizes increased anticipated eating enjoyment more strongly than experienced eating enjoyment. By coding Rating as 0 for experienced rating and 1 for anticipated ratings (rather than − 0.5 and 0.5 respectively), a spotlight analysis revealed that the effect of portion size on experienced eating enjoyment was not statistically significant (B = 0.16, *t* = 1.43, *p =* 0.15). As can be seen in Fig. 4a, other spotlight analyses revealed that the difference between anticipated and experienced ratings was statistically different for the smallest portion (B = -1.05, *t* = − 4.4, *p <* 0.001) and for the medium portion (B = -0.68, *t* = − 4.25, *p <* 0.001 (as shown in Table 3), but not for the largest portion (B = -0.13, *t* = − 0.5, *p =* 0.61), indicating that children underestimated how much they would actually enjoy the smaller two portions (but not the largest ones).

Table 3 further shows that eating enjoyment was lower for applesauce than for brownie (B = -1.29, *t* = − 7.91, *p <* 0.001) but none of the interactions involving food type were statistically significant (all *p*’s > 0.10). There was a significant interaction between portion size and initial hunger (*p* < 0.001), indicating that initial hunger increased the association between portion size and eating enjoyment. To illustrate this interaction effect, we plotted anticipated and experienced eating enjoyment in two groups of children, dichotomized via a median split on their average initial hunger over the seven sessions (see Fig. 4b). Among very hungry children, portion size increased eating enjoyment (B = 0.64, *t* = 5.69, *p <* 0.001), and did so similarly for anticipated and experienced ratings (interaction of portion size and rating: B = 0.36, *t* = 1.62, *p =* 0.11). For hungry children, portion size did not influence eating enjoyment (B = 0.03, *t* = 0.32, *p =* 0.75), and its effect was not different for anticipated and experienced ratings (interaction of portion size and rating: B = 0.38, *t* = 1.79, *p =* 0.07).

#### Perceived healthiness

**Fig. 5:**
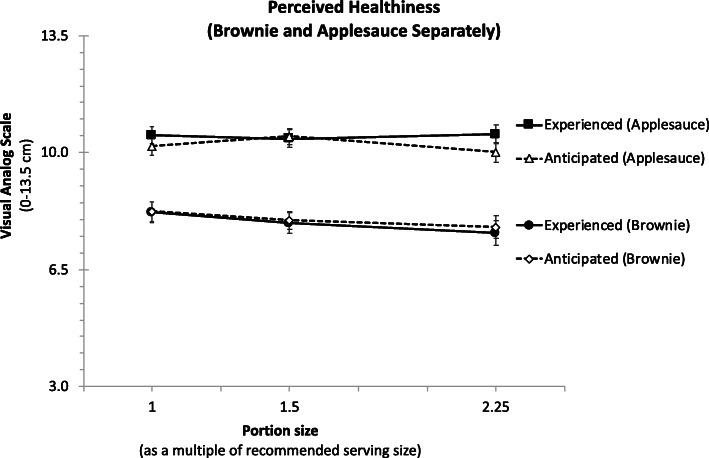
Means anticipated and experienced perceived healthiness, for both brownie and applesauce. The portion sizes represent the recommended serving size, or 1.5 or 2.25 multiple of this size respectively. Perceived healthiness is larger for applesauce than for brownie and is unaffected by portion size for both foods

The pattern of results was very different for perceived healthiness than for the other two ratings. As Fig. 5 shows, healthiness ratings were significantly higher for applesauce than for brownie (B = 2.50, *t* = 18.81, *p* < 0.001) and were barely affected by portion sizes (B = − 0.12, *t* = − 1.95, *p* = 0.051). None of the other coefficients was statistically significant (all *p*’s > 0.11).

## Discussion

The study produced four key results. The first was that the portion sizes of two common food snacks strongly influenced 8-to-11-year-old children’s ratings of hunger and eating enjoyment but not their perception of the healthiness of a portion. Children acknowledged that applesauce was healthier than brownie but judged that eating a 32 g (146 kcal) portion of brownie was not healthier than eating a 125% larger portion of 71 g (328 kcal). This is consistent with prior research showing that elementary school children determine health value based on food groups independently of portion size [38]. These results imply that health-based appeals are unlikely to nudge children to switch to age-appropriate portions, especially considering that smaller portions were judged much less satiating (based on changes in hunger from pre- to post-intake measurements) and less enjoyable to eat than larger portions. This is consistent with the findings of previous studies showing that, in children, enjoyment of food is the first driver of choice [39]. It corroborates the theory that eating pleasure is an effective lever of food choice for children [40].

The second key result is that portion size increased anticipated eating enjoyment but not experienced eating enjoyment. The eating enjoyment ratings provided by the smaller two portions (but not of the largest ones) were underestimated, that is, anticipated enjoyment was below experienced enjoyment for smaller portions. Initial hunger impacted the effects of portion size on eating enjoyment. Among very hungry children, both anticipated and experienced eating enjoyment increased with portion size. Among moderately hungry children, anticipated and experienced eating enjoyment were barely influenced by portion size. In contrast, portion size influenced anticipated and experienced ratings of residual hunger and of healthiness perceptions similarly. This suggests that, when judging portion sizes, children are not subject to mispredictions of hunger changes or of healthiness judgments, but underestimate enjoyment from smaller portions (but not larger ones). Children also fail to anticipate that – provided they are hungry rather than very hungry – eating a 32 g portion of brownie or a 100 g portion of applesauce will be just as enjoyable as eating a portion that is 125% larger. This suggests that helping children better anticipate enjoyment from smaller portion sizes may be a useful track to motivate them to accept and choose smaller snack portions.

The third key result was that, although children rated brownie as more enjoyable to eat than applesauce and rated applesauce as healthier than brownie, portion size influenced anticipated and experienced residual hunger similarly for both foods. In prior research in adults, the expected satiating effect or estimated calorie content of food increased linearly with energy density for foods with low energy density, whereas the relationships for high energy-dense foods reveal an underestimation [28]. Further research is necessary to determine if the discrepancy between our results and previous results regarding the role of calorie density comes from differences between the judgements of adults and children or from the fact that the cognitive processes involved in judging the satiating properties of different foods are not the same as those involved in judging the effects of increasing portion sizes on residual hunger of the same food.

The fourth and final key result was that, unlike previous studies conducted with adults, portion size linearly increased the anticipated eating enjoyment in children for both snacks. In contrast, a study by Cornil and Chandon [24] among adults found that the anticipated eating enjoyment for brownie increased until the middle portion and then decreased slightly, while the experienced eating enjoyment decreased linearly with portion size. This suggests that adults have the ability (unlike children) to accurately forecast that eating enjoyment ultimately decreases with quantity for these kinds of food. It may be that they rely more on cognitive rules when making food judgements than 8- to-11-year-old children, whose cognitive processes with regards to food and eating are still evolving [40, 41]. Of course, it would be of interest to explore this track by comparing children and adults’ judgments in a study specifically designed for that. The observation of a difference between children and adults may be related to the fact that the phenomenon of sensory-specific satiety (i.e. the decline of liking of a specific food over consumption) may manifest itself differently in children and adults [42]. For example, unlike among adults, sensory-specific satiety does not transfer to other foods with similar sensory characteristics in children [42]. Another explanation may be that the children in our study liked large portions of brownie more than the young Parisian women (average age 22 years old) in Cornil and Chandon’s study [24] who may have been more concerned with overeating. On average, the chosen brownie portion was 77% larger in the present study than in their study (298 kcal vs. 168 kcal).

The results of this study should be interpreted in the light of its limitations and strengths. In the absence of prior research on this topic, no power calculation could be run a priori. The sample size was quite small (although it is similar to the sample size used for other studies and reveals significant effects). In addition, contrary to the brownie, applesauce is amorphous so whether the children could distinguish the three portion sizes of applesauce easily as for brownie is a questionable point. However, our finding from the analysis over the group that children were able to predict their residual hunger as accurately for applesauce as for brownie suggests that the steps taken to facilitate the estimation of the portions served were successful.

By asking children to consume the portions served entirely, this study enabled the comparison of pre- and post-intake ratings for the same portion. Still, it would be interesting to explore the robustness of our findings in a different paradigm, where children would be asked to serve themselves, choose their desired portion size and eat ad libitum rather than choose among a selection of fixed portions. This would provide a more granular measure and avoid potential floor or ceiling effects. Future research should also examine apportionable foods [43] or foods sold in small pre-cut portions, such as candies, for which it is the number of units rather than the portion size that drives portion size perception and preferences (for example: [44]). Similarly, investigating the role of the shape of the portions could be interesting. In our study, the portions of brownie only increased in length and the portions of applesauce only in height. Earlier studies show that portions increasing along more than one dimension (e.g., in both width and length) appear to increase more slowly than those that increase in just one dimension [35].

Future studies should also include participants with different characteristics. Our study focused on children who were for the most part 9 or 10 years old; it would be interesting to look at children of different ages, both younger and older. Older children are more likely to also take into account social appeal (linked to the need to support their self-image when socializing) and taste when choosing unhealthy snacks [45]. In addition, given that social norms about portion size and eating habits may vary across different socio-economic groups and that SES is a strong predictor of weight status [46], investigating the same questions in deprived SES groups would be useful. Finally, it is often the parents, not the children themselves, who choose the size of the snacks, and they often disagree about what constitutes a small, medium, or large portion of food [47]. It would therefore be interesting to contrast the anticipations of parents and children regarding the effects of portion size on hunger relief, eating enjoyment, and healthiness judgements.

## Conclusion

Overall, this study advances the understanding of how 8-to-11-year-old children evaluate different portion sizes of familiar foods on hunger changes from pre- to post-intake, eating enjoyment, and healthiness, both before and after food intake. Children’s judgments revealed the strong impact of portion size on hunger changes from pre- to post-intake, its small impact on healthiness perception, and its positive impact on anticipated eating enjoyment. However, children did not anticipate the null effect of portion size on eating enjoyment and underestimated the eating enjoyment provided by small and moderate portions.

Studies on children’s portion size-related evaluations are too rare. The present study forms the groundwork for an area of research that will hopefully inform governmental agencies, catering services, food companies and caregivers when making portion sizes decisions for children.

## Supplementary information

**Additional file 1: Figure S1.** Study timeline (from session #1 to session #7 over 7 weeks) and photos of the 3 portion sizes offered for the brownie and the applesauce (presented in metal boxes with compartments and lids).

**Additional file 2: Table S1.** Separate mixed model regression coefficients (SE) for each food and each rating of the three dependent variables.

**Additional file 3: Analysis S1. **Comparison of children who completely vs. did not completely eat the portions. Choice of food portion made by the children at the end of the first session. Individual-level analyses of the effects of portion sizes on children’s anticipated and experienced ratings.

## Data Availability

The datasets generated and/or analyzed during the current study are not publicly available in accordance with the signed consent forms by parents but are available from the corresponding author on reasonable request.

## Abbreviation

BMI: Body Mass Index
