# Supplementary material for: Effects of snack portion size on anticipated and experienced hunger, eating enjoyment, and perceived healthiness among children
Source: Int J Behav Nutr Phys Act. 2020 Jun 1;17:70. doi: 10.1186/s12966-020-00974-z (PMC7268352; doi:10.1186/s12966-020-00974-z)
Supplement: Supplementary file 2 — Additional file 2: Table S1. Separate mixed model regression coefficients (SE) for each food and each rating of the three dependent variables. [file 12966_2020_974_MOESM2_ESM.pdf]

Supplemental Table 1. Separate mixed model regression coefficients (SE) for each food and each rating of the three dependent variables.

		**Residual Hunger**				**Eating Enjoyment**				**Perceived Healthiness**			
		Anticipated (pre-intake)		Experienced (post-intake)		Anticipated (pre-intake)		Experienced (post-intake)		Anticipated (pre-intake)		Experienced (post-intake)	
		B	SE	B	SE	B	SE	B	SE	B	SE	B	SE
**Brownie**	Intercept	8.65	0.26	8.12	0.19	10.25	0.20	10.81	0.18	8.09	0.24	7.99	0.30
	Portion size	-0.86**	0.12	-0.77**	0.12	0.58**	0.12	0.31*	0.13	-0.19	0.10	-0.26**	0.08
	Initial hunger$^{\Diamond}$	0.53**	0.10	0.80**	0.06	0.12	0.08	0.24**	0.06	0.18	0.09	0.05	0.05
	Portion size × Initial hunger$^{\Diamond}$	0.02	0.05	0.01	0.05	0.09*	0.04	0.24**	0.06	-0.02	0.04	0.03	0.04
	Female	-1.36*	0.52	-0.51	0.37	0.11	0.40	-0.63	0.37	-1.07*	0.49	-0.74	0.59
	Age$^{\Diamond}$	0.57	0.33	0.39	0.24	0.17	0.26	-0.25	0.24	0.19	0.32	-0.12	0.38
	z-BMI$^{\Diamond}$	-0.27	0.23	-0.11	0.16	-0.15	0.18	-0.24	0.16	-0.36	0.21	-0.56	0.26
**Applesauce**	Intercept	8.27	0.25	7.98	0.23	8.79	0.29	9.64	0.32	10.24	0.22	10.52	0.23
	Portion size	-0.77**	0.12	-0.63**	0.12	0.46**	0.12	-0.03	0.15	-0.07	0.10	0.04	0.09
	Initial hunger$^{\Diamond}$	0.60**	0.10	0.77**	0.07	0.09	0.12	0.02	0.10	0.17	0.09	0.07	0.06
	Portion size × Initial hunger$^{\Diamond}$	0.05	0.05	0.01	0.05	0.06	0.05	0.13*	0.07	-0.02	0.04	0.06	0.04
	Female	-0.14	0.51	-0.85	0.47	0.07	0.59	-0.99	0.64	0.02	0.44	-0.36	0.45
	Age$^{\Diamond}$	0.10	0.34	0.27	0.31	0.17	0.39	-0.27	0.43	0.18	0.29	-0.41	0.30
	z-BMI $^{\Diamond}$	0.27	0.22	-0.37	0.20	0.07	0.25	0.19	0.27	0.00	0.19	-0.09	0.19

Note: B=unstandardized coefficient of regression; all regressions controlled for child sex, age, and z-BMI. SE=Standard Error. $^{\Diamond}$ indicates that the variable was mean-centered; ** indicates that the coefficient is statistically different from zero at p<0.01 (* at p<0.05).

C. Schwartz, C. Lange, C. Hachefa, Y. Cornil, S. Nicklaus, P. Chandon. ***Effects of snack portion size on anticipated and experienced hunger, eating enjoyment, and perceived healthiness among children***
