# Supplementary material for: Effects of snack portion size on anticipated and experienced hunger, eating enjoyment, and perceived healthiness among children
Source: Int J Behav Nutr Phys Act. 2020 Jun 1;17:70. doi: 10.1186/s12966-020-00974-z (PMC7268352; doi:10.1186/s12966-020-00974-z)
Supplement: Supplementary file 3 — Additional file 3: Analysis S1. Comparison of children who completely vs. did not completely eat the portions. Choice of food portion made by the children at the end of the first session. Individual-level analyses of the effects of portion sizes on children’s anticipated and experienced ratings. [file 12966_2020_974_MOESM3_ESM.pdf]

Supplemental Analyses 1.

1. *Comparison of children who completely vs. did not completely eat the portions*

There were no leftovers (defined as more than 1 gram of food) in 77.3% of the cases (79.9% for brownie and 74.5% for applesauce, $\chi^2(1)$=2.04, *p*=0.16); Out of the 83 children, 27 children (32.5%) were categorized as completers, meaning that they never left more than one gram of food across the six eating occasions, and the rest (56 children, 67.5%) were categorized as non-completers.

Analyses of variance showed that non-completers (NC) were not statistically different from completers (C) on any of the control variables (hunger at the start of the session: M_{NC}=9.9 ± 2.4 vs. M_C=9.15 ± 3.0, *F*(1,82)=1.5, *p*=0.22; zBMI: M_{NC}=0.45 ± 1.2 vs. M_C=0.33 ± 1.1, *F*(1,82)=0.2, *p*=0.65; age: M_{NC}=9.4 ± .8 vs. M_C=9.5 ± .8, *F*(1,82)=0.3, *p*=0.61; percent female: M_{NC}=54% vs. M_C=56%, $\chi^2(1)$=0.03, *p*=0.87).

The two groups differed on one dependent variable, anticipated enjoyment (M_{NC}=9.4 ± 2.0 vs. M_C=10.3 ± 1.5, *F*(1,82)=4.7, *p*=0.03) but not on the other five (experienced enjoyment: M_{NC}=10.0 ± 2.1 vs. M_C=10.8 ± 1.7, *F*(1,82)=2.9, *p*=0.09; anticipated residual hunger: M_{NC}=8.0 ± 2.8 vs. M_C=8.9 ± 2.3, *F*(1,82)=1.8, *p*=0.19; experienced residual hunger: M_{NC}=7.7 ± 2.9 vs. M_C=8.5 ± 2.4, *F*(1,82)=1.9, *p*=0.18; anticipated healthiness: M_{NC}=9.0 ±1.8 vs. M_C=9.2 ± 1.8, *F*(1,82)=0.2, *p*=0.64; experienced healthiness: M_{NC}=9.1 ± 2.0 vs. M_C=9.3 ± 2.1, *F*(1,82)=0.1, *p*=0.80).

2. *Choice of food portion made by the children at the end of the first session.*

We examined the choice of food portion made by children at the end of the first session. Out of the 83 children who rated the brownies, 2 (2.4%) chose the smallest portion, 19 (22.9%) chose the middle portion and 62 (74.7%) chose the largest portion. Analyses of variance showed that chosen portions of brownies were rated lower in terms of residual hunger (M_{chosen}=7.5 ± 3.3 vs. $M_{not\ chosen}$=8.9 ± 3.5, *F*(1, 246)=9.1, *p*=0.003), higher in eating enjoyment (M_{chosen}=11.3 ± 1.7 vs. $M_{not\ chosen}$=9.9 ± 2.7, *F*(1, 245)=18.9, *p*<0.001), and similarly in terms of healthiness (M_{chosen}=8.0 ± 3.1 vs. $M_{not\ chosen}$=8.0 ± 2.6, *F*(1, 245)<0.01, *p*=0.99).

Out of the 77 children who rated the applesauce, 15 (19.5%) chose the smallest portion of applesauce, 27 (35.1%) chose the middle portion and 35 (45.5%) chose the largest portion. Analyses of variance showed that chosen portions of applesauce were rated similarly in terms

of residual hunger (M_{chosen}=8.1 ± 3.2 vs. $M_{not\ chosen}$=8.2 ± 3.3, F(1, 225)=0.05, p=0.83), higher in eating enjoyment (M_{chosen}=9.7 ± 2.8 vs. $M_{not\ chosen}$=8.5 ± 3.1, F(1, 229)=8.4, p=0.004), and similarly in terms of healthiness (M_{chosen}=10.5 ± 2.2 vs. $M_{not\ chosen}$=10.1 ± 2.4, F(1, 228)=2.1, p=0.15).

These results show that, unsurprisingly, children chose the portion that they anticipated to enjoy the most and that would leave them the least hungry and did not base their choice on the healthiness of the portion.

3. *Individual-level analyses of the effects of portion sizes on children's anticipated and experienced ratings*

Because effects found at the aggregate level when pooling across individuals might not occur at the individual level, we conducted separate individual-level regressions of the effects of portion size on the three ratings given by each child, for each dependent variable, both pre- and post-intake, and for each food. For example, given that 83 children rated their anticipated residual hunger after eating a small, medium, and large portion of brownie, we obtained 83 regression coefficients from 83 separate regressions with 3 observations per regression (one observation per portion size). We then distinguished whether each individual regression coefficient was positive or negative, indicating whether ratings increased or decreased with portion size. Through three logistic binary regressions, we examined whether the likelihood of positive coefficients varied with the timing of the ratings (pre vs. post-intake), the type of food, and their interaction. These logistic binary regressions also included initial hunger at the time of the session and were controlled for sex, age, and z-BMI.

Among the individual-level regressions of anticipated and experienced residual hunger, only 28% of the regression coefficients of portion size were positive. This means that most children (72%) anticipated and experienced decreasing residual hunger as portion sizes increased. This proportion was similar whether the ratings were pre-intake anticipations or measured after intake (Wald=0.55, p=0.46). However, this proportion was higher for applesauce (M=33%) than for brownie (M=22%, Wald=4.64, p=0.03), indicating that children expected portion sizes to decrease their residual hunger less for applesauce than for brownie.

For eating enjoyment, 62% of the coefficients were positive, indicating that eating enjoyment increased with portion size for most children. However, this proportion was larger pre-intake (M=68%) than post-intake (M=56%, Wald=5.39, p=0.02), which means that a

significant number of children failed to experience the increase in eating enjoyment that they were anticipating from larger portions. The proportion of positive coefficients was also larger for brownie (M=68%) than for applesauce (*M*=55%, Wald=5.48, *p*=0.02).

For perceived healthiness, the proportion of positive coefficients was 48%, indicating a lack of consensus about the direction of the effects of portion size on perceived healthiness, and this proportion was similar across foods and the timing of the rating. None of the interactions between rating timing (pre or post intake) and food product was statistically significant (*p's*>0.13).

Overall, the individual-level analyses confirmed the results of the aggregate analyses, albeit with larger differences between brownie and applesauce. For both products however, residual hunger decreased with portion size for the majority of children, and this proportion was the same for pre- and post-intake ratings. Second, eating enjoyment increased with portion size for the majority of children, but more so when it was measured before intake. Finally, children were about equally split about whether healthiness increased or decreased with portion size.
