# Supplementary material for: Effects of snack portion size on anticipated and experienced hunger, eating enjoyment, and perceived healthiness among children
Source: Int J Behav Nutr Phys Act. 2020 Jun 1;17:70. doi: 10.1186/s12966-020-00974-z (PMC7268352; doi:10.1186/s12966-020-00974-z)
Supplement: Supplementary file 1 — Additional file 1: Figure S1. Study timeline (from session #1 to session #7 over 7 weeks) and photos of the 3 portion sizes offered for the brownie and the applesauce (presented in metal boxes with compartments and lids). [file 12966_2020_974_MOESM1_ESM.pdf]

Supplemental Figure 1. Study timeline (from session #1 to session #7 over 7 weeks) and photos of the 3 portion sizes offered for the brownie and the applesauce (presented in metal boxes with compartments and lids)

Session #1	Session #2	Session #3	Session #4	Session #5	Session #6	Session #7
**Anticipated**	**Experienced**	**Experienced**	**Experienced**	**Experienced**	**Experienced**	**Experienced**
6 portions	1 portion	1 portion	1 portion	1 portion	1 portion	1 portion
counterbalanced order	counterbalanced order					

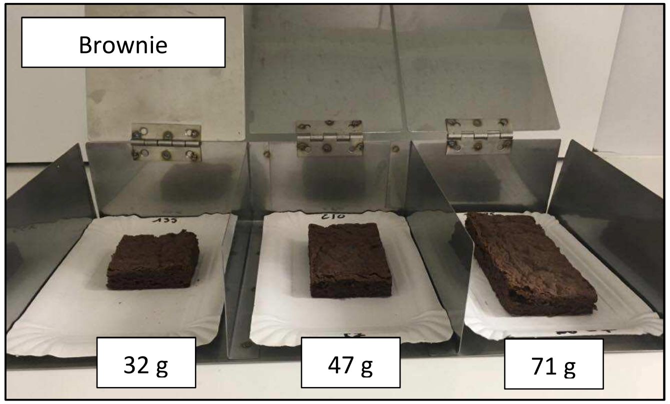

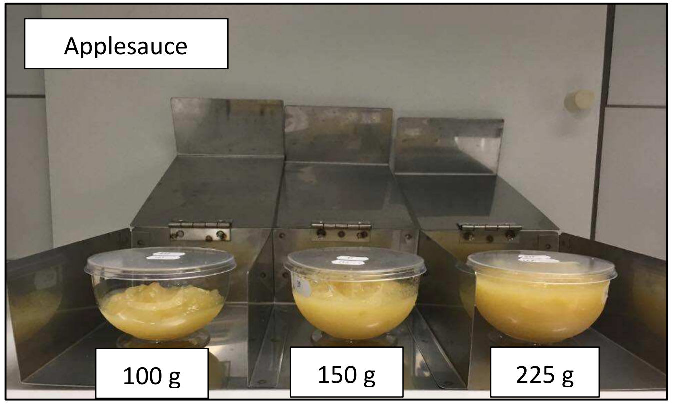

C. Schwartz, C. Lange, C. Hachefa, Y. Cornil, S. Nicklaus, P. Chandon. ***Effects of snack portion size on anticipated and experienced hunger, eating enjoyment, and perceived healthiness among children***
